# Epigenetics and the overhealing wound: the role of DNA methylation in fibrosis

**DOI:** 10.1186/s13069-015-0035-8

**Published:** 2015-10-01

**Authors:** Roisin Neary, Chris J. Watson, John A. Baugh

**Affiliations:** UCD School of Medicine and Medical Science, Conway Institute for Biomolecular and Biomedical Research, University College Dublin, Belfield, Dublin 4 Ireland

**Keywords:** Fibrosis, Fibroblast, Myofibroblast, DNA methylation, 5-azacytidine, 5-aza-2′-deoxycytidine

## Abstract

Fibrosis is a progressive and potentially fatal process that can occur in numerous organ systems. Characterised by the excessive deposition of extracellular matrix proteins such as collagens and fibronectin, fibrosis affects normal tissue architecture and impedes organ function. Although a considerable amount of research has focused on the mechanisms underlying disease pathogenesis, current therapeutic options do not directly target the pro-fibrotic process. As a result, there is a clear unmet clinical need to develop new agents. Novel findings implicate a role for epigenetic modifications contributing to the progression of fibrosis by alteration of gene expression profiles.

This review will focus on DNA methylation; its association with fibroblast differentiation and activation and the consequent buildup of fibrotic scar tissue. The potential use of therapies that modulate this epigenetic pathway for the treatment of fibrosis in several organ systems is also discussed.

## Review

### Introduction

Defined by the pathological accumulation of extracellular matrix (ECM) proteins, fibrosis results in scarring and thickening of the affected tissue. In essence, fibrosis is an exaggerated wound healing response which interferes with normal organ function.

**Fig. 1:**
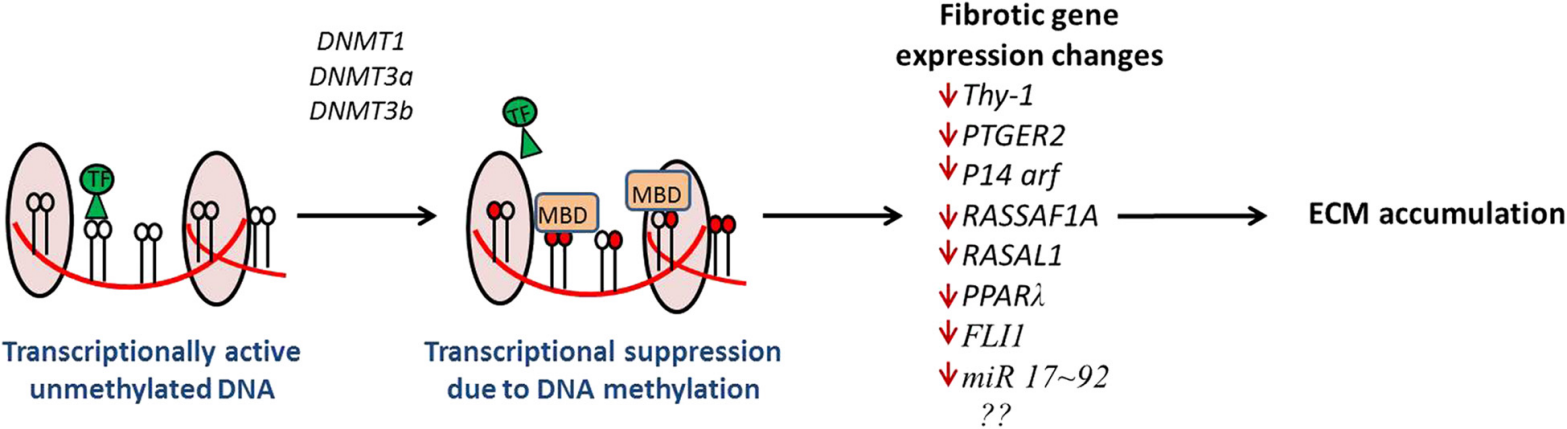
Epigenetic regulation of fibrotic gene expression profiles. The absence of DNA methylation permits gene transcription. DNA methylation, the addition of a methyl group to carbon 5 on the cytosine ring, is catalysed by the DNA methyltransferase enzymes. Methyl binding domain (MBD) proteins are recruited to methylated DNA and result in gene silencing by preventing transcription factor binding. Several genes associated with the development of fibrosis, highlighted in the figure, have been shown to undergo silencing as a result of DNA hypermethylation. It is likely that many other genes yet to be identified are also involved in the epigenetic regulation of fibrosis. These associations suggest the potential use of agents which target this methylating event for therapeutic use in fibrotic disease

Fibrosis is a characteristic feature of many chronic diseases that result in end-stage organ failure [1]. In the developed world, these fibrotic-driven diseases are a major cause of morbidity and mortality, accounting for 45 % of all deaths [2]. The primary pathways associated with tissue injury and the development of disease are relatively well-studied. Despite this, the precise molecular mechanisms underlying aberrant healing and fibrosis remain poorly understood. Effective anti-fibrotic therapies to prevent rapid disease progression are currently limited.

The process of tissue repair is complex. Tight regulation of ECM synthesis and degradation ensures normal tissue architecture. This necessary process can, however, lead to a progressive irreversible fibrotic response if tissue injury is severe/repetitive or if the wound healing response itself becomes deregulated [3].

Fibrosis has diverse etiologies where several factors can be associated with the progression of disease. Regardless of the initiating factor, all fibrotic diseases converge upon a final common pathway leading to fibroblast activation with resultant remodelling of the tissue [4].

### The fibroblast: wound healing or fibrosis

Fibroblasts are mesenchymal cells responsible for the synthesis of ECM components including collagens, fibronectin and elastin [5]. Under normal physiological conditions, fibroblasts are vital in maintaining the structural integrity of organs. In response to tissue injury, these cells are activated by pro-fibrotic cytokines and undergo a change in phenotype from normal relatively quiescent cells involved in slow turnover of ECM to highly proliferative and contractile myofibroblasts. Myofibroblasts, characterised by expression of the contractile protein alpha smooth muscle actin (αSMA), regulate connective tissue remodelling by combining the ECM synthesising features of fibroblasts with cytoskeletal characteristics of contractile smooth muscle cells [6–8]. In normal repair processes, the myofibroblast responses are diminished as the repair resolves to form a scar, occurring predominantly by apoptosis of these hyperactive cells or by reversion back to a more quiescent fibroblast phenotype [8-10]. During pathological fibrosis, however, these myofibroblasts persist in tissue resulting in fibrosis and hence impair normal organ functions.

Evidence suggests that myofibroblasts associated with disease pathogenesis have several origins with the major contribution arising from increased proliferation of resident fibroblasts. Several other sources have been suggested that are reviewed in detail by McAnulty [11].

Regardless of the origin of myofibroblasts, it appears that there is a persistent phenotypic change in the fibroblast inhibiting the apoptosis or reversion to a resting phenotype that occurs during the resolution phase of normal wound healing responses. This idea is supported by ex vivo work showing that fibroblasts isolated from fibrotic tissue maintain their hyperactive phenotype in the absence of a pro-fibrotic environment [12, 13].

Factors that promote fibroblast differentiation and activation, such as growth factors and cytokines, are well studied; however, the mechanisms that contribute to the maintenance of the pro-fibrotic myofibroblast phenotype are less understood. One mechanism that may account for the permanent hyperactive switch in fibroblasts is epigenetic modification of gene expression.

### Epigenetics

Epigenetics, the study of heritable changes in genome function that do not alter the nucleotide sequence, plays an important role in the regulation of gene expression. Modifications include DNA methylation, histone modifications and microRNA alterations. These events are crucial during early development, play a role in cell differentiation and have now also been implicated in disease pathogenesis [14, 15].

#### DNA methylation

DNA methylation occurs when the carbon 5 on the cytosine ring is methylated in a reaction catalysed by DNA methyltransferase (DNMT) enzymes, resulting in the formation of 5-methylcytosine (5MeC).

Methylation generally occurs on cytosine residues which precede a guanosine in the DNA sequence (the CpG dinucleotide). These CpGs are often found within proximal promoter regions and are known as CpG islands. Generally speaking, CpG island promoters are unmethylated and the absence of methylation is considered a permissive state for transcriptional activity. Upon methylation, the transcriptional activity of genes becomes suppressed and this is then passed on to daughter cells during the cell cycle.

Methylation and 5MeC formation results in transcriptional repression by one of two mechanisms (Fig. 1). Firstly, by direct interference, 5MeC formation can impede the specific binding of transcription factors to recognition sites in their respective promoters [16]. Several transcription factors, for example, the cyclic AMP-dependent activator CREB, E2F, HIF and NFkB, recognise sequences that contain CpG residues and binding of each has been shown to be inhibited by methylation [16]. Secondly, gene silencing may occur by direct binding of specific transcriptional repressors to methylated DNA. These methyl-CpG-binding proteins can exert regulation over the expression of multiple genes via their interaction with methylated DNA and their association with histone modifying enzymes [17]. MeCP1 and MeCP2 were the first two protein complexes identified. Following this, several new proteins including MBD1, MBD2 and MBD4 were described. For the most part, these repressor protein complexes bind methylated DNA through their methyl CpG binding domain (MBD) motif [16].

The relevance of CpG island promoter methylation and its association with disease development and progression was first observed in cancer, where DNA methylation silenced important tumour suppressors enabling cancer to progress [18]. The emerging role of DNA methylation in fibrosis will be discussed further below.

#### Demethylation

A relatively novel area of research associated with DNA methylation and disease pathogenesis, based upon the recent discovery of the ten-eleven translocation (TET) family of enzymes (TET1, TET2 and TET3), is now emerging. Whilst the DNMT family of enzymes establish and maintain DNA methylation, TET enzymes are implicated in DNA demethylation and epigenetic control of gene expression [19, 20]. Conventionally, 5MeC formation is associated with a transcriptionally repressed chromatin state whilst TET enzyme activity and subsequent demethylation enables gene transcription to proceed [21]. These enzymes convert 5MeC to 5-hydroxymethylcytosine (5hmC), which undergoes subsequent deamination and base excision repair by thymine DNA glycosylase [21]. 5MeC derivatives can then be recognised and removed by the base excision repair machinery resulting in the demethylation of once methylated cytosines. Much less is known about the epigenetic modification 5hmC, most likely accounted for by the difficulty in detecting both global and locus-specific levels; efficient techniques to achieve this have only been established in the previous 5 years [22]. The physiological significance of DNA oxidation in epigenetic regulation therefore remains poorly understood. With regard to disease, much like DNA methylation, most findings are associated with cancer development and progression [23, 24]. In the context of fibrosis, research is quite limited. Further work is required for understanding the role of TET proteins and 5hmC in gene regulation and disease.

#### Interaction of epigenetic modifications

It is becoming increasingly clear that DNA methylation plays a central role in directing histone modifications, and regulating micro RNA expression, and thus represents an important point of interaction between these epigenetic mechanisms.

Histone proteins are associated with DNA and are important for chromatin packaging and remodelling. The N-terminal tails of these proteins undergo post-translational modifications including acetylation, deacetylation and methylation which alter their interaction with DNA and can thus impact gene expression. Although much is still to be learned, the relationship between DNA methylation and histone modifications is becoming increasingly clear. It is now believed that the two mechanisms cooperate in controlling gene expression. It seems that the relationship can work in both directions: histone methylation can help to direct DNA methylation patterns, and DNA methylation may serve as a template for some histone modifications after DNA replication. The link between the two alterations may be partially mediated through methyl-binding proteins such as MeCPs or MBDs which are capable of recruiting histone deacetylases to the methylated region [25].

A bi-directional relationship between DNA methylation and non-coding RNAs (ncRNA) has also recently been highlighted. ncRNAs are functional molecules transcribed from DNA but not translated into proteins. In general, these molecules function to regulate gene expression at the transcriptional and post-transcriptional level. ncRNAs that appear to be involved in epigenetic processes are divided into two main groups: the short nc-RNAs (<30 nts) and the long nc-RNAs (> 200nts). MicroRNAs (miRNA) are perhaps the most well known of the regulatory ncRNA classes and have been shown to play a role in heterochromatin formation, DNA methylation targeting and gene silencing. miRNAs comprise a class of short ncRNAs, 18–25 nucleotides in length, which regulate gene expression post-transcriptionally. These miRNAs bind to the 3′UTR of their target genes and control gene expression by translational suppression and/or destabilisation and degradation of the target gene. Interestingly, each miRNA is predicted to have many targets, and each mRNA may be regulated by more than one miRNA [26]. Epigenetic mechanisms, including DNA methylation and histone modifications regulate the expression of some miRNAs. Conversely, another subset of miRNAs can control the expression of important epigenetic regulators, including the DNA methyltransferase and histone deacetylase enzymes. This complicated network of feedback between miRNAs and epigenetic pathways appears to form an epigenetics, miRNA regulatory circuit, and is important in organising the whole gene expression profile. Disruption of this circuit interferes with normal, physiological functions and can contribute to disease processes [26, 27].

A clear example of the relationship between DNA methylation and miRNA expression occurs rather interestingly in a study looking at pulmonary fibrosis. Dakhlallah et al. highlight a miRNA-DNMT regulatory circuit. This work identified reduced expression of miR17~92 which was associated with increased DNMT-1 and a pro-fibrotic phenotype. The authors showed that several miRNAs from the miR17~92 cluster targeted DNMT-1 expression resulting in a negative feedback loop [28].

Given these associations, it is likely that multiple interactions involving several epigenetic modifications contribute to the pathogenesis of many diseases.

### Epigenetics and disease

The role of epigenetics in disease is perhaps most well documented in the case of cancer whereby DNA hypermethylation and subsequent silencing of tumour suppressor genes allows rapid disease progression. Although much less common, hypomethylation can also occur activating the aberrant expression of oncogenes [29–31]. DNA hypomethylation due to alterations in the demethylation machinery have been identified in cancer. TET enzymes are mutated in several types of cancer, affecting their activity and likely altering genomic 5hmC and 5MeC patterns [24].

Histone modifications and microRNA alterations have also been implicated in numerous disease processes [32]. Similar to DNA methylation, these are most evident in the setting of cancer.

Given these pathological associations, inhibiting epigenetic processes has become apparent as a potential therapeutic target. Epigenetic therapies are now used as treatment options in some cancers. Inhibitors of DNA methylation as well as histone deacetylase inhibitors (vorinostat and romidepsin) have been FDA approved for the treatment of certain malignancies. Although an miRNA targeting agent has yet to be approved, this field of research is expanding exponentially. miRNAs have not only shown promise in the field of drug development but have also shown potential as effective biomarkers in a number of different clinical scenarios [33].

#### Inhibiting DNA methylation

5-azacytidine (5-aza; Vidaza) and 5-aza-2-deoxycytidine (5-azadC; Decitabine) are demethylating agents that are effective for the treatment of myelodysplasic syndromes (MDS) and acute myeloid leukaemia (AML) [34, 35]. These compounds are internalised by cells and incorporated into DNA. Covalent adducts with cellular DNMT1 are formed thereby depleting enzyme activity and inhibiting methylation of DNA during cell division [36].

Re-expression of epigenetically silenced tumour suppressor genes is a rational strategy for the treatment of human neoplasms with these epigenetic modifiers. Nonetheless, the mechanism of action behind their clinical efficacy remains unclear. Ongoing clinical trials are attempting to identify tumour suppressor genes that upon re-expression can induce remission and cure in patients. On the other hand, the pleiotropic biological effects of DNMT inhibitors and recent reports demonstrating lack of association between clinical response and methylation reversal of candidate tumour suppressor genes, suggest a complex mechanism behind their clinical efficacy that may involve a cytotoxic effect [37, 38].

Although these compounds have demonstrated beneficial clinical effects, there are some drawbacks relating to their use. Epigenetic modifications, such as DNA methylation, are important in normal physiology; thus, inhibiting this process is likely to yield some unwanted side effects. Adverse effects such as nausea, vomiting, diarrhoea and loss of appetite have been observed. Myelosuppression is another major side effect that occurs upon treatment with 5-aza/5-azadC. Most patients recover within a 5–6-week period; however, this does limit dose and duration of treatment [39].

It is also apparent that the half-life of DNA demethylating agents is quite short; hence, the development of 5-aza therapeutic strategies for the treatment of fibrotic pathologies as opposed to blood malignancies would require investigations into drug formulations which could improve efficacy as an anti-fibrotic agent. Such approaches that could be explored include controlled release preparations and pro-drug formulation approaches to optimise bioavailability and increase the therapeutic window for the hypomethylation indication. Unlike in cancer, a sustained low-dose release may be more beneficial for the treatment of fibrosis, avoiding high cytotoxic doses. Furthermore, it would be of value if new formulations could be devised that enable specific organs to be targeted depending on the fibrotic pathology. Some of these approaches are being pursued, including oral formulations [40, 41] and targetable drug loaded biodegradable microspheres [42–44].

Whilst aberrant epigenetic modifications are most widely studied in the context of cancer, they have been identified to play a role in the development of several other disease pathologies. Recent reports highlight a growing link between epigenetic modifications and fibrosis. The approval of epigenetic drugs in cancer holds promise for the use of these agents as effective therapeutics for the treatment of several different diseases. Although in vitro and in vivo work on the associations between epigenetic modifications and the pathogenesis of fibrosis are constantly emerging, as of yet, there are no clinically approved epigenetic therapies to treat fibrotic diseases. Given that DNA methylation is the principal focus of this review, we will go on to describe the associations and role of this alteration in the progression of fibrosis in the following section.

### DNA methylation and fibrosis

It has been hypothesised that maintenance of the activated state of the myofibroblast during fibrotic disease reflects a failure to return to its resting state as in normal physiological wound healing. During the process of differentiation, active myofibroblasts acquire significant changes in their gene expression profiles [45]. Whilst various environmental features within the injured tissue are likely to be involved in promoting fibroblast differentiation, it is proposed that stable changes in gene expression due to chromatin modifications are responsible for the sustained myofibroblast phenotype [46]. Understanding the epigenetic mechanisms by which fibroblasts acquire these pro-fibrotic phenotypes and subsequently differentiate to myofibroblasts is important for complete elucidation of the pathogenesis of fibrosis as well as its management and treatment.

#### Pulmonary fibrosis

A role for DNA methylation in regulating fibroblast phenotype during disease progression is supported in several studies. Gene-specific hypermethylation events as well as global changes in DNA methylation have been identified in a number of organ systems. Two genomic studies comparing the profile of lungs from normal and idiopathic pulmonary fibrosis (IPF) patients revealed extensive DNA methylation changes, thus implicating DNA methylation in the control of IPF lung gene expression [47, 48]. It is also important to consider the fact that epigenetic modifications may not be homogeneous across organ fibroblast populations and in some fibrotic pathologies; it has been acknowledged that sub-populations of fibroblasts exist characterised by distinct cellular phenotypes. This is particularly well documented in the lung where fibroblasts, depending on their expression of certain genes, have more or less potential to differentiate into highly proliferative, contractile myofibroblasts. One candidate gene that is implicated in the epigenetic control of fibroblast phenotype is thymocyte differentiation antigen-1 (Thy-1). Thy-1 has been implicated in controlling the activation of the latent form of the pro-fibrotic growth factor transforming growth factor beta 1 (TGF-β1) and represents a potentially important anti-fibrotic gene [49, 50]. Diminished Thy-1 expression is associated with a more fully differentiated myofibroblast phenotype, potentially due to exaggerated TGF-β1 signalling, and fibrotic tissue buildup [45], [51]. Loss of Thy-1 is observed in lung tissue from patients with IPF where reduced expression of Thy-1 in active myofibroblast clusters, known as fibroblastic foci, was shown to be due to promoter methylation. Reduced expression of this anti-fibrotic gene was one of the first demonstrations of the importance of epigenetics in fibrosis [52]. Interestingly, a study by Robinson et al. showed that hypoxia, which has previously been implicated in the pathogenesis of pulmonary fibrosis, promotes global DNA hypermethylation as well as *Thy*-*1* gene-specific hypermethylation in primary human lung fibroblasts. *Thy-1* gene expression was suppressed in these hypoxic fibroblasts but could be restored by treatment with the DNMT inhibitor 5aza-2′deoxycytidine (5azadC) [53].

Prostaglandin E receptor 2 expression (PTGER2) has also been linked with pulmonary fibrosis. Work by Huang et al. showed that IPF fibroblasts were resistant to the anti-fibrotic effects of prostaglandin E_2._ The authors suggested that this may be due to the loss of *PTGER2* as a result of hypermethylation-induced silencing. This was demonstrated in both IPF fibroblasts and in fibroblasts from a bleomycin mouse model of fibrosis. Increased global DNA methylation was also observed [54].

The persistence of activated myofibroblasts during the progression of fibrotic responses may be accounted for by resistance to apoptosis. Epigenetic mechanisms may play a role in mediating the anti-apoptotic properties of pro-fibrotic myofibroblasts. *P14*^*ARF*^ induces cell cycle arrest. Hypermethylation and subsequent silencing of *P14*^*ARF*^ was shown in IPF patient-derived fibroblasts. This hypermethylation-induced silencing event may contribute to pathological lung fibrosis as myofibroblasts may acquire an anti-apoptotic phenotype. Myofibroblasts can then persist in tissue causing an excess production of ECM [55].

As previously mentioned, a recent exciting study by Dakhlallah et al*.* looking at both lung biopsies and fibroblasts from IPF patients as well as a bleomycin-induced pulmonary fibrosis model that highlights a novel epigenetic regulatory circuit involving DNMT enzymes and a miRNA cluster. This work identified elevated expression of *DNMT1* which was associated with diminished expression of a miRNA cluster and a pro-fibrotic phenotype. Interestingly, in this study, and in all cases described above, treatment with the DNA methylation inhibitors 5-azacytidine (5aza) or 5-azadC restored expression of the miRNA or of the gene in question and reduced fibrosis.

#### Renal fibrosis

DNA methylation has also been implicated in the pathogenesis of renal fibrosis. A genome-wide study investigating cytosine methylation patterns in healthy and chronic kidney disease patient samples identified significant differences. A core set of genes known to be related to kidney fibrosis, including those encoding collagens, showed cytosine methylation changes correlating with downstream transcript levels, thus implicating a role for epigenetic dysregulation in chronic kidney disease development [56].

In a separate study by Bechtel et al., a gene-specific hypermethylation event was eluded to in the case of Ras GTPase activating-like protein 1 (RASAL1). *RASAL1* expression was decreased in the kidneys of a folic-acid induced fibrotic mouse model. This hypermethylation-induced silencing of *RASAL1* was associated with increased DNMT1 expression. In support of this, DNMT1^+/−^ mice when compared with wild-type controls exhibited reduced renal fibrosis when challenged with folic acid. The DNA methylation inhibitor 5-aza also displayed beneficial effects in the kidney where the fibrotic fibroblast phenotype was normalised in vitro and experimental murine renal fibrosis ameliorated [57].

The research mentioned thus far implicates DNA methylation in the fibrotic response and alludes to the potential use of DNMT inhibitors as viable therapeutics. It is interesting to note that associations between DNA demethylation, TET enzyme activity and fibrosis have also been uncovered. A recent study by Tampe et al. alludes to a potential novel role for demethylation and TET enzymes in the treatment of renal fibrogenesis. In this study, renal fibrosis was again associated with *RASAL1* hypermethylation and subsequent suppression. Interestingly, this work also demonstrated loss of *TET3* expression during disease progression. Application of BMP7, which has endogenous anti-fibrotic effects through TGF-β1 antagonism, to pro-fibrotic renal fibroblasts was associated with induction of *TET3*, normalisation of *RASAL1* promoter methylation and restored *RASAL1* expression [58]. This work reveals a new mechanism which may be exploited to facilitate therapeutic DNA demethylation to reverse kidney fibrosis.

#### Liver fibrosis

Interestingly, *RASAL1* methylation has also been correlated with fibroblast differentiation in rat hepatic stellate cells. Hypermethylation of RASAL1 was associated with the perpetuation of fibroblast activation and fibrogenesis in the liver. Treatment with 5-azadC reduced fibroblast proliferation and restored *RASAL1* expression [46]. This work by Tao et al. also suggests an additional epigenetic control mechanism of fibrosis.

As alluded to previously, recruitment of proteins which bind methylated DNA affects gene expression. Although slightly contradictory, MeCP2, has now been shown to play a pivotal role in the development of fibrosis and fibroblast differentiation. During liver fibrosis, hepatic stellate cells (HSCs) become activated and undergo myofibroblast transdifferentiation; expression of MeCP2 is altered during this process. Induction of MeCP2 during HSC activation contributes to the loss of expression of several anti-fibrotic mediators including *RASAL1* and peroxisome proliferator-activated receptor gamma (PPARλ) [59, 60]. These findings are backed up by in vivo studies. In a rat model of hepatic fibrosis, MeCP2 expression was increased compared with healthy controls [46]. In a separate in vivo study looking at pulmonary fibrosis, myofibroblast differentiation was attenuated in MeCP2^−/−^ mice [61]. This work similarly suggests a positive relationship between MeCP2 expression and the development of fibrosis, but the authors of this study focus on pro-fibrotic αSMA expression. Loss of MeCP2 in this case resulted in decreased expression of the myofibroblast marker in both mouse lung fibroblasts and in vivo in a bleomycin murine model of pulmonary fibrosis. The authors in this paper link reduced MeCP2 with reduced fibrosis due to pro-fibrotic (αSMA) gene silencing. Whilst both studies support a pro-fibrotic function for MeCP2, these data suggest an alternate mechanism to the work carried out by Tao et al. who associate reduced MeCP2 with reduced methylation, hence transcriptional activation of anti-fibrotic *RASAL1/PPARλ*. These findings suggest that the effects of transcriptional repression by MeCP2 may be gene-/species-dependent or that perhaps complete knockout of MeCP2 has knock-on effects on other associated pathways which may contribute to the effects on αSMA expression.

In addition to the gene-specific methylation events highlighted above, work examining the genome-wide DNA methylation status in early stage liver fibrosis has also been carried out. Although the aforementioned work primarily implicates DNA hypermethylation in the development of a fibrotic phenotype, rather interestingly early stage liver disease was associated with global DNA hypomethylation [62]. Comparing control and early stage fibrotic livers, the authors’ identified reduced genome-wide DNA methylation in a mouse model of carbon tetrachloride-induced fibrosis. Furthermore, a gene-specific hypomethylation event in the case of secreted phosphoprotein1 (Spp1) gene, a known inducer of inflammation, was also alluded to. These results suggest that DNA hypomethylation may be crucial for the onset and initial triggering of liver fibrosis. Hypomethylation and the resultant increased expression of genes involved in the initiation of fibrosis, such as Spp1, may precede the onset of liver fibrosis. The authors suggest the potential involvement of a switch from hypo- to hypermethylation during the development and progression of liver fibrosis.

#### Cardiac fibrosis

Very recent reports now implicate a role for the epigenetic regulation of cardiac fibrosis. A study by Watson et al. identified a role for DNA methylation in hypoxia-induced cardiac fibrosis. Fibroblast exposure to hypoxic conditions upregulated collagen and αSMA which was associated with increased global hypermethylation and increased expression of the DNMT enzymes DNMT1 and DNMT3B via the hypoxic response transcription factor HIF-1α. These data were complemented by in vivo evidence of robust fibrosis in areas of hypoxic myocardium in human cardiac tissue samples. In addition to this, application of the DNA methylation inhibitor 5-azadC reduced the hypoxia-induced activation of myofibroblasts in vitro [63]. A separate study by Tao et al. also alludes to a role for DNA methylation in the pathogenesis of cardiac fibrosis. Increased expression of DNMT3a was observed in activated cardiac fibroblasts and in fibroblasts isolated from isoprenaline-treated rats. Elevated expression of the methylating enzyme was associated with increased expression of αSMA and silencing of the tumour suppressor gene RASSAF1A. Therapeutic intervention with 5-aza reduced fibroblast expression of pro-fibrotic markers and restored RASSAF1A gene expression.

Several murine models of cardiac disease now also support a pathogenic role for DNA methylation in myocardial fibrosis and highlight beneficial effects upon therapeutic intervention with inhibitors of this epigenetic process.

A study looking at the mechanisms of cadmium-induced cardiac dysfunction identified beneficial effects of 5-azadC. Male mice were exposed to cadmium for 4 weeks with or without the DNA methylation inhibitor. Cadmium exposure led to interstitial fibrosis and collagen 1 deposition which was reduced upon intervention with 5-azadC [64]. In addition, separate work showed that angiotensin II-induced cardiac fibrosis was reduced upon administration of 5-aza [65]. Treatment with 5-aza prevented the accumulation of myocardial collagen in this model. The spontaneously hypertensive rat has been shown to demonstrate significant cardiac fibrosis. In a novel study by Watson et al., intervention with 5-aza reduced both total and perivascular collagen deposition in the SHR [66], again highlighting protective effects of the epigenetic modifying agent and suggesting a potential causal role for heightened methylation in the fibrotic heart.

Rather interestingly, Zeisburg et al. have recently identified a gene-specific hypermethylation event in the case of RASAL1 in cardiac fibrosis (as is evident in both liver and renal fibrosis). This work demonstrated aberrant DNA promoter methylation and subsequent transcriptional silencing of RASAL1 which enhanced endothelial-mesenchymal transition (EndMT) and myocardial fibrosis. Furthermore, the authors report that endothelial cells in the heart possess an intrinsic mechanism to reverse aberrant DNA promoter methylation involving TET3-mediated hydroxymethylation. Administration of anti-fibrotic BMP7 therapeutically induced TET3-mediated reversal of RASAL1 [67]. These novel findings provide further proof-in-principle evidence that aberrant DNA methylation contributes to cardiac fibrosis and that demethylation may serve as anti-fibrotic therapeutic strategy in chronic heart disease in the future.

Whilst the work highlighted above demonstrates protective effects of inhibiting DNA methylation in several experimental models of cardiac fibrosis, global levels of DNA methylation have not been assessed. Interestingly, this has somewhat been addressed in human studies of both heart failure and cardiomyopathies where differential patterns of DNA methylation have been identified [68, 69]. Although this data is not specific to cardiac fibrosis, these pathologies are somewhat associated with the excessive accumulation of extracellular matrix proteins and thus suggest the potential for a role of an altered DNA methylation profile driving fibrotic responses in the myocardium. This requires further investigation.

#### Scleroderma

A role for DNA methylation has also been implicated in scleroderma (systemic sclerosis, SSc). Scleroderma is a complex multi-system disorder associated with vascular damage and auto-immunity, ultimately resulting in fibroblast activation and collagen accumulation in the skin and internal organs. Genome-wide DNA methylation analysis in dermal fibroblasts from patients with systemic sclerosis identified several differentially methylated genes comparison to healthy matched controls. Differential DNA methylation patterns were evident in several genes/pathways known to be associated with fibrogenesis. These included genes encoding collagen proteins as well as those involved in the TGFβ1/integrin and Wnt/β-catenin signalling pathways. Moreover, DNA methylation status was shown to correlate with gene expression in the majority of genes evaluated in this study. For the most part, differentially methylated pro-fibrotic genes displayed hypomethylated profiles; hence, their expression was increased in SSc compared to controls [70].

In addition to this work which highlights genome-wide DNA methylation changes in systemic sclerosis, a separate study looked more specifically at epigenetic regulation of enhanced collagen expression in scleroderma fibroblasts. Elevated collagen levels were associated with increased levels of the DNMT enzymes and methyl binding proteins in dermal fibroblasts isolated from patients with systemic sclerosis compared to controls. The authors first showed that addition of 5-azadC normalised collagen levels in scleroderma fibroblasts. Following on from this, a mechanism responsible for increased collagen production via an epigenetic repressive effect on the collagen suppressor gene *FLI1*, was identified. Through methylation-induced silencing of *FLI1*, the repressive effect on collagen synthesis was removed, thus permitting increased collagen gene transcription [71].

Interestingly, alterations in DNA methylation have also been highlighted in fibroblasts isolated from keloids. Keloids can occur as a result of an overactive dermal wound healing response. A study using ChIP-Chip analysis and DNMT inhibitor treatment also allude to an epigenetically altered programme in fibroblasts isolated from keloid scars when compared to those isolated from healthy scar tissue [72].

In light of the abovementioned work highlighting associations between DNA methylation and a fibrotic phenotype in several organ systems, together with the beneficial in vitro and in vivo effects of 5-aza/5-azadC, it seems plausible to suggest that epigenetic modifying drugs such DNA methylation inhibitors may demonstrate significant clinical efficacy in treating fibrosis. Of note, however, these drugs which have displayed anti-fibrotic properties target enzymes on a global level and may bring about adverse side effects; therefore, caution must be exerted. Interestingly, in some of the fibrotic conditions highlighted above, the increase in DNA methylation has been attributed to specific upregulation of either the maintenance enzyme DNMT1 or the de novo methyltransferases DNMT3a and DNMT3b. Although one might expect an increase in DNMT3a or DNMT3b to be primarily involved in initiating pro-fibrotic DNA methylation patterns, studies in the liver, lung, heart, kidneys and systemic sclerosis have implicated a role for elevated DNMT1 and/or DNMT3 or DNMT3b in fibrosis [28, 47, 57, 71, 73–75]. These important observations further highlight the link between dysregulation of DNMTs and fibrosis and the rationale of targeting them therapeutically. The current DNMT inhibitors in clinical practice 5-aza and 5-azadC preferentially target DNMT1 and thus affect the maintenance of DNA methylation. However, it could be of great value to develop DNMT inhibitors that are specific to de novo methylating enzymes, given that this enzyme sub-type is likely responsible for generating new additional methylation marks in the development of fibrosis. Using an inhibitor to DNMT3a or DNMT3b rather than DNMT1 may also reduce the likelihood of adverse side effects as the de novo methylating enzyme levels should be minimally expressed in most mammalian adult tissue types. Given the complex roles of the DNMT enzymes in initiating and maintaining disease-relevant DNA methylation patterns, it is likely that a combination therapy targeting both the maintenance and the de novo methyltransferases would be most beneficial. Treating early in disease with a global DNMT inhibitor could allow pathological cells to reset their normal DNA methylation pattern but might be associated with a higher risk of side effects. These side effects could perhaps be minimised by then maintaining patients on therapies that specifically target the de novo DNMTs and do not affect the maintenance of DNA methylation patterns in healthy tissues. Given that histone modifications and microRNA changes appear to interact with DNA methylation, it may be the case that a combination of epigenetic therapies would be more efficacious. Although much progress has been made, this area certainly warrants further investigation into the gene-specific alterations that occur in fibrotic tissue during disease progression.

### A role for hypoxia in the fibrotic response

As previously alluded to, although the final common pathway in fibrosis appears to be similar across organ systems, the initiation event may vary. One recurring theme contributing to the pathogenesis of fibrotic disease is hypoxia. Hypoxia occurs when a cell’s demand for oxygen exceeds supply and can lead to the accumulation of gene expression changes.

Associations between hypoxia, fibrosis and epigenetics are becoming ever apparent. It is likely that cellular responses to acute hypoxia, which may occur as a consequence of tissue ischemia or increased oxygen consumption, play a crucial role in shaping the wound healing response. Consistent with a role as a fibrogenic stimulus, hypoxia stimulates fibroblast proliferation and induces aSMA expression. This corresponds with increased collagen synthesis in human renal fibroblasts, pulmonary fibroblasts, cardiac fibroblasts and hepatic stellate cells [53, 63, 76]. The effects of hypoxia are predominantly described as pathological, resulting from the hyperproliferation of fibroblasts and excessive ECM deposition. In some cases, however, as is evident in the heart post-myocardial infarction, hypoxia-induced activation of fibroblasts can have protective effects. Fibroblast activation and subsequent myocardial remodelling is required here to ensure restoration of normal cardiac structure and function [77].

One mechanism by which hypoxia may influence the fibroblast phenotype is via the regulation of DNA methylation [78]. A role for hypoxia modulating the DNA methylation profile of cells has been described and is suggested to arise from hypoxic regulation of DNMT expression [79–81]. Interestingly, there are putative hypoxia responsive elements (HRE) on the promoters of all three active DNMTs with in vitro data indicating that hypoxia-induced DNMT1 and DNMT3b promoter activity is HRE-dependent [63]. As mentioned earlier, studies in fibroblasts from both the lungs and the heart have demonstrated hypoxic regulation of DNA methylation. Robinson et al. showed that culturing human pulmonary fibroblasts under hypoxic conditions results in a hypermethylated phenotype, and this was associated with methylation-induced silencing of anti-fibrotic *Thy-1* [53]. Further supporting a role for hypoxia induced methylation, work by Watson et al. identified increase gene and protein expression of both DNMT1 and DNMT3b in hypoxic human ventricular fibroblasts [63].

Another role for hypoxia in the fibrotic response arises from its effects on angiogenesis, although the outcome of this is very much organ-dependent. Hypoxia can promote vessel growth by upregulating multiple pro-angiogenic pathways [82]. In addition to this, several genes which play important roles in angiogenesis, including *EPO* and *VEGF*, are regulated by *HIF-1α.* The precise relationship between angiogenesis and fibrosis remains controversial as considerable evidence supports both a positive and negative regulatory role for angiogenesis in progressive fibrosis. An association between excessive angiogenesis and fibrosis in IPF has been suggested based on both human studies and bleomycin-induced murine pulmonary fibrosis [83, 84]. The development of liver fibrosis is also accompanied by increased angiogenesis [85]. On the other hand, inadequate angiogenesis has been implicated in a model of interstitial renal fibrosis. In this model, decreased renal microvascular density is accompanied with progressive renal impairment, excessive matrix deposition and interstitial fibrosis [86]. Similarly, reduced extent of microvasculature is associated with cardiac fibrosis development [87]*.* Given the need to maintain adequate tissue perfusion and oxygenation, and the negative impact of fibrosis on this process, it is likely that hypoxic regulation of epigenetic mechanisms such as DNA methylation are a central but complex feature of the wound healing response and progression to fibrotic disease.

### Novel perspectives

There has been an array of additional work highlighting a role for DNA methylation in the pathogenesis of fibrosis. Although very interesting, literature in some of these areas is still somewhat limited. We have therefore decided to include the following topics in this perspectives section to draw attention to the work that is being done in this exciting field of research.

#### Influence of DNA methylation on heritable susceptibility to disease

Whilst most of the examples mentioned thus far indicate a role for DNA methylation in the suppression of anti-fibrotic gene expression during the progression of disease, it is interesting to note that there may also be a role for epigenetics in protecting offspring from fibrotic disease.

The concept that epigenetic signatures can be inherited in an intergenerational fashion is supported by recent experimental studies. Alterations in the methylation of germ cell DNA caused by in utero exposure to environmental toxins mediate the transgenerational transmission of adult-onset pathologies in multiple organs [88]. Researchers from a recent study also reported that feeding adult male rats a high-fat diet led to insulin resistance in female offspring that was associated with β cell dysfunction, and this was linked with gene hypomethylation [89]. Furthermore, feeding a low-protein diet to male mice altered the global CpG methylation patterns in their offspring, and these changes were associated with adaptation of hepatic lipid and cholesterol metabolism [90].

A link between inherited epigenetic signatures and fibrosis has now also been made by Mann et al*.* who report a multi-generational epigenetic adaptation of hepatic wound healing. A role for DNA methylation in the control of both anti- and pro-fibrotic gene expression is identified. It was shown that the offspring of rats with liver fibrosis have reduced fibroblast activation and that these protective effects are due to an epigenetic suppressive mechanism, suggesting that remodelling of DNA methylation underpins these gene expression adaptations. Increased expression of anti-fibrotic *PPARλ* was shown to be associated with promoter hypomethylation whilst simultaneous promoter hypermethylation induced silencing of the pro-fibrotic growth factor *TGF-β1.* The adaptive effect on fibrogenesis was apparent within a single generation but was more pronounced when liver fibrosis was present in the successive F0 and F1 generations, which is suggestive of a cumulative process [91]. Intergenerational downregulation of liver fibrogenesis may therefore have the biological advantage of ensuring greater fitness of subsequent generations that are exposed to the environmental pressures of potent liver toxins.

Interestingly, another recent discovery has identified a link between maternal transmission of DNA methylation at the promoters of specific circadian clock genes and the development of hepatic fibrosis [92]. The circadian clock controls, among others, daily metabolism and subsequent activity rhythms. Rhythmicity is driven by a circadian timing system composed of a central pacemaker and master oscillator, the suprachiasmatic nucleus (SCN), which can also drive self-sustained oscillators in peripheral cells and tissues [93]. Transcription factors, which drive the expression of their own negative regulators, are central to the molecular rhythmicity of SCN neurons and oscillating cells [94]. This transcriptional feedback loop is regulated by complex mechanisms, including post-translational modifications of circadian proteins, which both maintain clock timing and enable adjustments to the clock machinery based on changes in the environment [95, 96]. Interestingly, epigenetics have also been shown to play an important role in the transcriptional activation of clock gene machinery, which relies on chromatin remodelling [97]—detailed discussion on this topic is beyond the scope of this review.

Returning to the subject of maternal transmission of altered DNA methylation patterns, and their subsequent contribution to fibrosis, a study by Mouralidarane et al*.* showed that offspring from obese mothers displayed disrupted daily expression of both *α-SMA* and *TGF-β1*. Peak expression of both pro-fibrotic markers was observed predominantly close to or in the dark phase—the period of maximal rodent activity. The authors alluded to the fact that repair and regeneration, which normally occur during rest cycles (i.e. during the day), were compounded in the activity period, and this may have enhanced the development of fibrosis. They therefore suggest that the development of hepatic fibrosis in offspring from obese mothers may arise from desynchronisation of normal rhythmic expressions of these fibrosis-inducing genes. Further experiments noted that the deregulated expression *α-SMA* and *TGF-β1* was associated with altered gene expression of two genes involved in circadian clock homeostasis, namely *BMAL1* and *Per2*. DNA methylation analysis at the promoter region of both genes identified a hypermethylated profile, which may account for the corresponding changes in *BMAL1* and *Per2* gene expression. This altered circadian clock gene expression, as a result of promoter hypermethylation, may in turn affect the disrupted daily expression of genes involved in fibrogenesis thus resulting in the development of a fibrotic phenotype.

In addition to this work, a growing body of evidence highlights an association between DNA methylation, circadian rhythm and the development of cancer. As mentioned above, epigenetics and chromatin remodelling play a role in controlling the transcription of clock gene machinery. Interestingly, a reciprocal relationship has also been uncovered whereby the clock can impart some control on DNA methylation—cells with a dysfunctional clock system demonstrate aberrant 5-methylcytosine expression [98]. It is well-established that circadian disruption results in substantial health consequences, numerous studies now indicating that shift workers suffer a higher incidence of cancer (reviewed in [99]). DNA hypermethylation has been linked with several cancers and interestingly studies now show aberrant DNA methylation patterns in every core clock gene in a variety of malignancies [100–103] Together, these findings suggest the existence of a pathogenic loop consisting of circadian rhythm disruption, altered DNA methylation and the development of cancer. Whether or not these changes in methylation are a cause or an effect of circadian rhythm deregulation is still unknown. This observation, together with the study highlighted above demonstrating maternal transmission of circadian gene hypermethylation and hepatic fibrosis, suggests the potential for a similar disordered circuit to exist and contribute to the pathogenic wound healing response. Alterations in genes involved in circadian rhythm homeostasis may lead to DNA hypermethylation (or vice versa), with the subsequent manifestation of organ fibrosis. This intriguing area of research certainly warrants further in-depth investigation.

#### Epigenetic control of macrophage phenotype

Although many of the studies highlighted in this review primarily focus on the direct role of epigenetics in controlling the myofibroblast phenotype, it is also emerging that epigenetic regulation of inflammatory cells such as macrophages may contribute to the pro-fibrotic environment. Macrophages not only play a key role in response to injury but also coordinate the wound healing response by controlling fibroblast activation. Macrophages are the main source of the pro-fibrotic growth factor TGF-β1 which drives fibroblast differentiation and ECM production [104]. In physiological wound healing, macrophages are cleared from the site of injury via apoptosis, but in fibrotic tissue, sustained macrophage activation may play an important role in sustaining myofibroblast activation via the exaggerated production of TGF-β1. Specifically, the early inflammatory response is driven by M1 macrophages that are important in clearing infection and necrotic tissue whilst the wound healing response is modulated by M2 macrophages [105]. In the context of fibrosis, exaggerated tissue injury may result from excessive M1 activation, whilst inappropriate M2 activation may drive exaggerated myofibroblast responses.

A relatively novel area of research regarding the epigenetic regulation of macrophage polarisation and its potential contribution towards fibrosis is emerging in the context of cardiac fibrosis. A recent article by Kim et al. alludes to this. The study identifies a pro-inflammatory role for M1 macrophages, whilst suggesting that the M2 type play a role in coordinating the fibrotic response. In a rat myocardial infarction model, treatment with the DNA methylation inhibitor 5-aza increased the number of M2 macrophages whilst simultaneously decreasing the number of M1 macrophages. This elevation of M2 type cells was associated with improved left ventricular function and a reduction of cardiac fibrosis [65]. Given that M2 macrophages are a source of TGF-β1 caution should be exerted when interpreting these findings. This macrophage phenotype appears to play more of a modulatory role in wound healing, and these potential contradictory roles of macrophages may arise due to the ability of these inflammatory cells to assume a wide spectrum of functional phenotypes determined by their microenvironment [106]. Macrophages appear to be important orchestrators and effectors of tissue repair, altering their function and phenotype to meet the needs of the healing tissue [105]*.*

This work does, however, highlight an important link between epigenetics and macrophage differentiation. In addition to this, it identifies a potential pathological pro-fibrotic contribution of DNA methylation through its effects on macrophages and that inhibiting this process with 5-aza can reverse these effects.

The suggestion that macrophage phenotype is controlled by epigenetics is further supported by work on adipose tissue. Obesity is associated with a switch from alternatively activated anti-inflammatory M2 macrophages to classically activated pro-inflammatory M1 type. Interestingly, results demonstrate that the switch from M2 to M1 in ob/ob mice is correlated with an increase in DNMT3B expression [107]. They also showed that DNMT3b knockdown is sufficient to induce M2 macrophage polarisation. Furthermore, knockdown of DNMT3b had important physiological effects in improving adipocyte insulin signalling.

Taken together, these findings suggest a relatively novel mechanism of epigenetic control of fibrosis through macrophage polarisation.

## Conclusions

Collectively, these findings strongly support the idea that alterations in DNA methylation have a substantial impact on fibroblast phenotype and promote the differentiation to pathological, scar-forming myofibroblasts. Novel findings also implicate DNA methylation in shaping the inflammatory phase of the wound healing response and influencing progression to fibrotic disease. Various components of the methylation machinery pose as potential therapeutic targets and refinement of such novel approaches will be achieved through increasing our understanding into the gene-specific modifications that occur during the pathogenesis of fibrotic diseases.

## Abbreviations

5aza: 5-azacytidine
5azadC: 5-aza-2-deoxycytidine
5hMC: 5-hydroxy-methylcytosine
5MeC: 5-methylcytosine
AML: acute myeloid leukaemia
DNMT: DNA methyltransferase
ECM: extracelluar matrix
HRE: hypoxic response element
HSC: hepatic stellate cell
IPF: idiopathic pulmonary fibrosis
MBD: methyl binding protein
MDS: myelodysplasic syndromes
MeCP: methyl-CpG-binding proteins
miRNA: microRNA
PPARλ: peroxisome proliferator-activated receptor gamma
PTGER2: prostaglandin E receptor 2
RASAL1: Ras GTPase activating-like protein 1
TET: ten-eleven translocation
TGF-β1: transforming growth factor beta
Thy-1: thymocyte differentiation antigen-1
αSMA: alpha smooth muscle actin
